# Recent Advances in Proteomic Studies of Adipose Tissues and Adipocytes

**DOI:** 10.3390/ijms16034581

**Published:** 2015-02-27

**Authors:** Eun Young Kim, Won Kon Kim, Kyoung-Jin Oh, Baek Soo Han, Sang Chul Lee, Kwang-Hee Bae

**Affiliations:** 1 Functional Genomics Research Center, KRIBB, Daejeon 305-806, Korea; E-Mails: issuekey@kribb.re.kr (E.Y.K.); wkkim@kribb.re.kr (W.K.K.); kjoh80@kribb.re.kr (K.-J.O.); bshan@kribb.re.kr (B.S.H.); 2 Department of Functional Genomics, University of Science and Technology of Korea, Daejeon 305-806, Korea

**Keywords:** adipocytes, adipogenesis, adipose tissue, browning, proteomics

## Abstract

Obesity is a chronic disease that is associated with significantly increased levels of risk of a number of metabolic disorders. Despite these enhanced health risks, the worldwide prevalence of obesity has increased dramatically over the past few decades. Obesity is caused by the accumulation of an abnormal amount of body fat in adipose tissue, which is composed mostly of adipocytes. Thus, a deeper understanding of the regulation mechanism of adipose tissue and/or adipocytes can provide a clue for overcoming obesity-related metabolic diseases. In this review, we describe recent advances in the study of adipose tissue and/or adipocytes, focusing on proteomic approaches. In addition, we suggest future research directions for proteomic studies which may lead to novel treatments of obesity and obesity-related diseases.

## 1. Introduction

Proteomics refers to the large-scale cataloging of proteins in a given cell, tissue or organism, and the proteome is the entire set of proteins. Essentially, the genome of the organism is nearly constant, whereas the proteome shows various patterns according to the cell type and the responses to various stimuli. Furthermore, the number of different proteins is expected to grow tremendously mainly due to alternative splicing and post-translational modifications (PTMs). Currently, over 80,000 alternative splice variants and at least 200 different PTMs are estimated or reported. Therefore, proteomics can cover fields, which cannot be approached by genomics. At present, proteomics is commonly used as a tool for identifying unique bio-signatures and novel biomarkers and for the mining of interaction partners and druggable targets. Thus, many scientists consider proteomics as a bridge between genomics and biology [1,2,3,4].

Obesity no longer refers only to being overweight. The World Health Organization (WHO) has officially recognized obesity as a chronic disease. Obesity is linked to health risks and can lead to various metabolic disorders, such as type II diabetes, cardiovascular disease, hypertension and certain cancers. The fundamental cause of obesity is an energy imbalance between energy input and output. Generally, this may due to the increased intake of energy-dense foods and decreased physical activity. Consequently, long-term energy imbalance induces the accumulation of an abnormal amount of body fat in white adipose tissue, which is composed mostly of white adipocytes [5,6].

There are two different types of adipose tissue, white adipose tissue (WAT) and brown adipose tissue (BAT). These adipose tissues have different colors, morphologies, functions, and biochemical features. BAT was known as a fat depot in rodents and newborn human. BAT oxidizes and dissipates excess energy as heat. Because BAT was recently discovered in adult humans and is correlated inversely with obesity, it has gained a considerable amount of attention with regard to efforts to overcome obesity by burning excess energy. On the other hand, WAT is the dominant type of adipose tissue in the human body, and it is distributed throughout the body. It functions primarily to store excess energy in the form of triglycerides (TGs). As a person’s weight increases, WAT is expanded by both increased adipocyte size (hypertrophy) and increased adipocyte numbers (hyperplasia) [7,8,9].

Recently, a new type of brown-like adipocyte with distinct gene expression patterns was discovered [10,11,12]. These novel brown-like cells, which reside within WAT, have been termed beige/brite adipocytes, or inducible brown adipocytes. They are induced under certain stimuli, such as exposure to cold. This induction of the brown adipocyte-like phenotype in WAT is called “browning”, through which the beige/brite cells in WAT are able to transform. These beige/brite cells are distributed throughout the human body and are highly activated in response to a variety of stimuli, including cold and the presence of endogenous hormones. Thus, WAT browning as well as BAT activation may contribute to a crucial strategy for overcoming obesity.

Collectively, a deeper understanding of the biological mechanisms that regulate the functions of adipose tissues or adipocytes will help with the development of novel strategies for suppressing obesity. To realize this, various proteomic approaches has been applied. The first proteomic approach was utilized in 1979 [13]. Changes in the protein expression patterns of 3T3-L1 cells during adipogenic differentiation were reported by using 2-DE analysis. Despite the early start to proteomic studies of adipose tissues or adipocytes, an extensive combined analysis of obesity and proteomics has not been conducted since that report [14,15]. However, a number of researchers have become interested in proteomic approaches involving adipose tissues and/or adipocytes due to the recent advances in proteomics technology and the discovery of beige/brite cells.

The aim of this review is to highlight the novel progress obtained using proteomic approaches with regard to adipose tissues and adipocytes. Additionally, future directions of proteomic approaches related to obesity are suggested.

## 2. Proteomic Studies of WAT

Proteomic analyses of tissue samples are demanding due to the unique characteristics of samples, such as heterogeneity and the complexity of the sample preparation process. For adipose tissues, the high lipid contents added an additional and difficult hurdle. Hence, there are relatively few proteomic analyses of adipose tissues. Nonetheless, to understand the functional and pathological roles of WAT, several rodent and human model systems have been constructed and applied to proteomic analyses [16]. Thus far, proteomic analyses have mainly been performed using samples from mouse, rat, pig, cattle and human WAT.

Proteome analyses of WAT provide meaningful clues with which to understand the molecular mechanisms of obesity and their roles in the pathogenesis of obesity-related diseases [17]. In particular, WAT was long considered as an inert organ, but now it is recognized as a highly active endocrine organ with numerous important physiological and pathological functions. Classically, WAT is classified into subcutaneous (sWAT) and visceral (vWAT) depots. sWAT, located under the skin, is widely distributed and acts as an insulator against heat or cold. On the other hand, vWAT, or abdominal fat, is located around internal organs (surrounding the omentum, gut and kidney), and is strongly associated with obesity-related metabolic disorders. Most proteomic studies have been carried out on these two types of adipose tissue.

### 2.1. Proteomic Analyses of Human WAT

Proteome profiling analyses of WAT from healthy human and/or obese patients have been done in an effort to elucidate the molecular mechanisms of the pathogenesis of obesity or obesity-related diseases. Xie *et al.* [18] characterized the human adipocyte proteome from sWAT of healthy, lean subjects, and identified a total of 1493 proteins. From proteome results, they suggested an important role of adipocyte mitochondria. Murri *et al.* [19] presented data obtained from a proteomic analysis of vWAT in pre-obese patients with type II diabetes as compared to pre-obese subjects showing normal glucose tolerance levels. Kim *et al.* [20] also reported protein-profiling data of vWAT which suggested that it was linked to the early pathogenesis of type II diabetes mellitus. They performed an analysis using samples from drug-naïve early type II diabetes mellitus and subjects with normal glucose tolerance levels. A total of 4707 proteins were identified, and 444 and 328 proteins were increased and decreased, respectively, in patients with type II diabetes mellitus. Recently, the effects of androgen, a sex hormone, on human sWAT and vWAT were assessed at the proteome level [21]. Researchers obtained WAT samples from 21 morbidly obese patients (seven males and seven females showing no evidence of androgen excess), and seven hyperandrogenic woman with polycystic ovary syndrome during bariatric surgery. Through an extensive 2D-DIGE analysis, they found similar proteome patterns between females with excess androgen and males, suggesting that androgens influence the function of adipose tissue. Brambilla *et al.* reported the shortgun protein profile of human WAT and its changes in relation to systemic amyloidosis [22]. They used the MudPIT proteome approach and compared protein profiles of human amyloid-affected WAT from patients and control counterparts. This result provides a clue with which to understand the molecular mechanisms of amyloidosis at the tissue level and ultimately to understand protein-folding diseases. To identify the proteins associated with gestational diabetes in omental adipose tissue, proteomic analyses using 2D-DIGE were carried out, after which the proteins involved in inflammation, lipid and glucose metabolism, and oxidative stress were identified as differentially expressed proteins [23]. Perez-Perez *et al.* [24] also reported the results obtained by comparative proteomic analysis of human omental adipose tissue, and they suggested several proteins such as transketolase and aminoacylase-1 as proteins involved in pathophysiology of obesity. Brambilla *et al.* published the proteome profiling data of sWAT in patients with transthyretin amyloidosis, compared to controls and patients with other types of amyloidosis [25]. Capobianco *et al.* performed the miRNA and protein expression analysis of vWAT from patients with severe obesity. They found two miRNA/protein targets (miRNA-141/YWHAG and miRNA-520e/RAB11A) and confirmed the functional interaction between these miRNAs and their target sequences on the corresponding mRNAs. They concluded that these miRNA/protein target pairs might be key players in the obese phenotype [26]. Recently, Mardinoglu *et al.* [27] combined data from RNA-seq and antibody-based immunohistochemistry to show the normal physiology of human WAT, and mined WAT-specific genes via comparing WAT to 26 other human tissues. Additionally, they identified several obesity-related metabolic changes on the basis of the analysis of sWAT transcriptomics and plasma metabolomics data. Through these approaches, they observed reduced glutaminolysis and alterations in the cytosolic branched-chain amino acids (BCAAs) metabolism in sWAT of obese subjects compared to lean subjects. Corton *et al.* [28] reported the protein expression profiles of omental adipose tissue biopsies obtained from morbidly obese women with or without polycystic ovary syndrome (PCOS) to examine the possible involvement of visceral adiposity in the development of PCOS. Although more detailed functional studies are needed, they revealed the several proteins displaying differential expression pattern in PCOS patients.

Overall, a lot of target genes have been identified and pathophysiological mechanisms of obesity and obesity-related diseases have been partially elucidated by proteomic approaches. However, it is desperately necessary to more detailed studies of candidate target genes, such as (tissue-specific) knockout experiment and regulation test via chemicals with high specificity to understand and overcome the obesity and its related diseases.

### 2.2. Proteomic Analyses of WAT in Disease Models

Proteomic analyses of WAT in disease models have been performed in order to elucidate the molecular mechanisms of the pathogenesis of this type of tissue. The proteomic analyses of human WAT provide a global, systematic and comprehensive approaches for investigate the pathophysiological mechanisms of obesity. However, proteomic analyses of human WAT remains problematic mainly due to use of WATs derived from patients with extremely heterogeneous life styles and genetic backgrounds. Thus, suitable animal models that allow genetic background and environmental conditions to be controlled are very useful in studies of obesity and/or its related diseases.

Peinado *et al.* undertook the adipose tissue protein profiling of Zmpste24^−/−^ mice, a model of lipodystrophy and premature aging [29]. Their data revealed major changes in the mitochondria function and in vimentin processing. Proteome changes after a treatment with bitter melon seed oil (BMSO) was also reported [30]. BMSO is rich in *cis*-9, *trans*-11, and *trans*-13 conjugated linolenic acid, and attenuates body fat deposition in high-fat-diet-induced obese C57BL/6J mice. A proteomic analysis of the C57BL/6J adipose tissue treated BMSO was conducted to acquire insight into how WAT is affected by BMSO and to explore the mechanism of the anti-adiposity effect of BMSO. Through proteomic data, it was suggested that the anti-adiposity effect of BMSO is associated with WAT delipidation, inflammation, and browning. These proteomic analyses of WAT of disease models provided clues for understanding the molecular mechanisms of obesity-related disease pathogenesis.

### 2.3. Proteomic Analysis of WAT during Caloric Restriction or Exercise

The proteome modulation of male Wistar rat WAT by aging and caloric restriction was monitored [31]. The results of the analysis of the proteome data suggested that the effects of caloric restriction on WAT are only weakly related to those of aging. Additionally, the proteome data provides insight into the mechanisms underlying the caloric restriction effects and clues for the discovery of novel biomarkers of aging. Leggate *et al.* analyzed proteomic changes in plasma and WAT after high-intensity intermittent training to determine if two weeks of high-intensity intermittent training altered inflammatory status in plasma and WAT in overweight and obese males [32]. The proteome change data suggest that two weeks of high-intensity intermittent training is sufficient to induce beneficial alterations. Although more detailed studies are needed, these proteomic results offer insight for scientists to understand the molecular mechanisms that affect caloric restriction or exercise.

### 2.4. Secretome Analysis of WAT

As mentioned above, adipose tissues are a key endocrine organ affecting whole-body energy homeostasis by secreting a variety of molecules, including adipokines. Thus, for an in-depth understanding of the pathogenic mechanisms of obesity, a secretome analysis of adipose tissue is of great interest [33,34,35,36,37,38]. Roca-Rivada *et al.* performed a secretome analysis of rat WAT explants from different anatomical localizations (visceral, subcutaneous, and gonadal fat). They constructed reference maps for location-specific adipose tissue secretomes and listed the 45 spots showing the most significant differences. Although these results could not provide the detailed differential protein networks or signal pathways, it was strongly suggested the differential role of adipose depending on its anatomical localization [34]. In a proteomic approach using SELDI-TOF, an analysis of the secretome of human vWAT was performed [35]. Chen *et al.* carried out 2-D LC-MS/MS and quantitative proteomic approaches using the adipose tissue of obese Zucker rats compared to that of lean rats to characterize obese adipose secretory proteins upon treatment with thiazolidinedione (TZD), a PPARγ agonist [36]. Lim *et al.* analyzed the secreted proteome of differentiated adipocytes and identified 97 and 203 secreted proteins in immortalized and primary rodent adipocytes, respectively [37]. In addition, they reported more than 80 N-linked glycosylation sites on adipocytokines and suggested a central role of the *O*-GlcNAc modification of intracellular proteins in the onset of insulin resistance [37]. Recently, they also reported quantitative secretome and glycome analyses of primary human adipocytes [38]. Chiefly due to the technical limitation of proteomic approaches, including sensitivity of mass spectrometer, the key secreted molecules involved in energy homeostasis have not been completely identified until now. However, we anticipate that a lot of key secreted molecules will be discovered by proteomic approaches as technical improvement could be continuously proceeded in the foreseeable future.

### 2.5. Proteomic Analysis of WAT in the Agricultural Sciences

In the agricultural sciences, a deeper understanding of the biology of adipose tissue is also essential, because adiposity is closely related to the quality of meat products. Using the 2D-DIGE system, Gondret *et al.* compared the proteomes of French Basque (BQ) pigs, which have a high potential for subcutaneous fat deposition, and the Large White (LW) modern lean-type pig breed [39]. They showed that the adipose tissue proteome in weaned piglets of the fat-type breed were similarity to those in obese humans in how they had, for instance, a more active metabolic cycle of triacylglycerols, low-grade inflammation, and lower cell apoptosis. Although proteomic studies using samples derived from livestock have not been extensively carried out, the need for these types of analyses of the adipose tissues of livestock has greatly increased.

### 2.6. Comparative Analysis of sWAT and vWAT Proteomics

The difference at the proteome level between sWAT and vWAT is a one of the major concerns, which interests many researchers. Perez-Perez *et al.* reported the results of a comparative proteomic analysis of the omental and subcutaneous adipose tissues of healthy obese patients by 2D-DIGE and MS analyses [40]. Through this study, 43 differentially expressed proteins were identified and the omental adipose tissue was suggested to have a high level of metabolic activity. It was also reported in a proteomic analysis of mature adipocytes and stromal vascular fraction (SVF) cells of vWAT and sWAT derived from healthy humans that WAT contributes to the major differences between subcutaneous and visceral fat depots [41]. Insenser *et al.* also published data obtained by a comparative proteomic analysis of sWAT and vWAT samples from six morbidly obese patients and six non-obese persons [42]. Through a 2D-DIGE analysis, they identified various proteins that may be involved in the development of obesity-related adipose tissue dysfunction in humans. Among the identified proteins, they were interested in osteoglycin, a protein that has been linked to cardiovascular dysfunction. This protein is up-regulated in obesity and shows different abundance levels in sWAT and vWAT [42]. Restelli *et al.* recently reported the comparative proteomic results between sWAT and vWAT of goat, focusing on proteins involved in immune and inflammatory response [43]. From these comparative proteomic analyses, the differences between sWAT and vWAT with regard to their physiological and pathological roles were gradually determined. In addition, sWAT and vWAT are sub-classified as depending on the anatomical locations. Thus, more detailed proteomic analyses of sub-classified types of sWAT and vWAT will be anticipated in the future.

## 3. Proteomic Analysis of White Adipocytes Using Stem Cells or Model Cell Lines

To obtain the proteomic data and further elucidate the molecular mechanisms of white adipocyte differentiation and the pathogenic mechanism of obesity-associated disorders, including insulin resistance and type II diabetes, several rodent and human model cell systems have been used. Among these, the mouse 3T3-L1 cell line and human adipose-derived stem cells are the typical models for adipocyte biology and adipocyte differentiation.

### 3.1. Proteomic Analysis for Adipocyte Biology Including Adipogenesis

Using human adipose-derived stem cells as an *in vitro* model of adipogenesis, DeLany *et al.* reported more than 50 proteins, which showed differentially expressed patterns (by more than two-fold). Among them, they focused on several heat-shock proteins (HSP20, HSP27, and HSP60) and suggested their involvement in adipogenesis [44]. Lee *et al.* also published proteome data during the adipogenic differentiation of human mesenchymal stem cells using 2-DE analysis [45]. They reported a total of 32 proteins, which displayed differential expression patterns during adipogenesis. Stapleton *et al.* investigated a glycogen-associated proteome in 3T3-L1 adipocytes to explain the roles of adipocyte glycogen [46]. They found several proteins, including 14-3-3-proteins, RACK1 and protein phosphatase 1 as glycogen-interacting proteins. These data could provide new insight into the regulation of the glycogen-bound proteins that are associated with the maintenance, organization and localization of glycogen particles in adipocytes. Kim *et al.* has been especially interested in biological phenomena related to lipid rafts. They performed a proteome analysis of adipocyte lipid rafts in 3T3-L1 adipocytes and suggested that gC1qR plays essential roles in adipogenesis and insulin signal transduction [47,48]. Kheterpal *et al.* performed a 2D-DIGE analysis of human sWAT from multiple depots (the arm and abdomen) after separation into stromal-vascular fraction (SVF) cells and mature adipocytes [49]. Approximately 200 proteins showing two-fold up- or down-regulation patterns were identified. The pathways related to cytoskeletal, glycogenic, glycolytic, lipid metabolic, and oxidative stress were differentially regulated between the mature adipocytes and SVF cells. Wilson-Fritch *et al.* reported a link between mitochondria biogenesis and white adipogenesis using combined proteomic and genomic strategies [50]. Additionally, they examined changes in the mitochondria composition during 3T3-L1 adipogenesis in response to a rosiglitazone treatment. To identify the proteins associated with insulin resistance in adipocytes, a comparative proteomic analysis using the NBS method was carried out in 3T3-L1 cells in which insulin resistance was induced by treatment with a TNF-α or dexamethasone [51]. After excluding already known adipokines and validating other candidates using immunoblot analyses, they finally focused on progranulin. Through various *in vitro* and *in vivo* experiments, they suggested that progranulin is a key adipokine that mediates high-fat-diet-induced insulin resistance and obesity via the secretion of IL-6 in adipose tissue. Thus, progranulin may be a valuable therapeutic target for treating obesity and obesity-related metabolic diseases. There have been several reports based on 2-DE analysis during the adipogenic differentiation of 3T3-L1 cells [52,53,54,55]. In 2009, the adipocyte proteome during differentiation was reported by *in vivo* labeling using a five-plex SILAC-based strategy [56]. The nuclear proteome and secretome were monitored during the course of 3T3-L1 adipocyte differentiation using four different heavy stable isotope forms of arginine. Through this method, a number of proteins that had not been documented previously were reported as differentially expressed proteins during adipogenesis. A proteomic approach using iTRAQ-coupled 2D LC-MS/MS was also applied during 3T3-L1 adipocyte differentiation [57]. Approximately 1000 protein species were quantitated with 106 proteins found which were significantly altered during differentiation. Additionally, the involvement of pyruvate carboxylase (PCX) and voltage-dependent anion-selective channel protein 2 (VDAC2) in adipogenesis was validated using RNAi experiments. Newton *et al.* reported the results of a proteomic analysis of 3T3-L1 adipocyte mitochondria during differentiation and lipid drop enlargement [58]. Mitochondria proteins at different stages of differentiation were harvested and labeled using the iTRAQ 8-plex kit. A total of 123 proteins showing significant changes in expression were listed, suggested that adipocytes enter an active metabolic state (e.g., increased flux through the TCA cycle and enhanced fatty acid oxidation). Through these approaches, various candidate proteins involved in the pathogenesis of obesity as well as adipogenesis were identified and validated. The data obtained from proteomic approaches substantially help us to understand the adipocyte biology and white adipogenesis.

### 3.2. Proteomic Analysis of Adipocyte Secretome and Membrane Proteome

Chiellini *et al.* investigated the human mesenchymal stem cell secretome at the early stages of adipocyte and osteoblast differentiation [59]. Mesenchymal stem cells can differentiate into adipocytes and osteoblasts. Using one-dimensional electrophoresis and tandem MS, they identified 73 proteins at day 0 and day 3 of adipocyte and osteoblast differentiation. PAI-1 was suggested as a regulator of the adipocyte/osteoblast balance. In addition, Kratchmarova *et al.* utilized systematic proteomic approaches to isolate and identify novel secreted factors that are differentially expressed during the adipogenic differentiation of 3T3-L1 cells into mature adipocytes [60]. Using 1-DE combined with nano-electrospray tandem mass spectrometry, they reported several secreted proteins, including pigment epithelium-derived factor and neutrophil gelatinase-associated lipocalin, which were involved in differentiation processes. Secretome and membrane proteome analyses were also carried out by several research groups [61,62,63,64,65]. Wang *et al.* obtained secretome data from a 2-DE analysis of a culture medium during the differentiation of 3T3-L1 cells [61]. Jeong *et al.* reported membrane proteomic data gained which was obtained the adipogenesis of human mesenchymal stromal cells by gel-based and non-gel-based approaches after subcellular fractionation [62]. Zhong *et al.* reported the temporal profiling of the secretome and nuclear proteome during the adipogenesis of primary human preadipocytes using iTRAQ-based quantitative proteomics [63]. They identified and quantified 420 proteins from the secretome of differentiated human adipocytes and reported a number of proteins whose differential expressions had not been previously documented during adipogenesis. Furthermore, they utilized antibody arrays to quantify changes in the levels of 182 adipokines during adipogenesis. Zyonic *et al.* also examined the secretome of primary cultures of human subcutaneous adipose-derived stem cells as an *in vitro* model of adipogenesis [64]. They analyzed conditioned media derived from four individual female donors after culturing in undifferentiated or adipogenic differentiated conditions. Approximately 80 proteins showing more than twice the number of relative differences were listed, with a particular emphasis on serine protease inhibitors (serpins). Lehr *et al.* also conducted complementary protein profiling of a concentrated conditioned medium derived from differentiated primary human adipocytes [65]. Through this analysis, 44 proteins were identified as novel adipokines. They also confirmed the regulation and secretion of these adipokines in primary human adipocytes and in serum and adipose tissue biopsies from obese patients and normal controls. The release and expression of complementary factor H, αB-crystallin, a cartilage intermediate-layer protein, and heme oxygenase-1 in adipocytes are controlled by differentiation and stimuli as well as by obesity. A proteomic analysis of these substances (also known as “adipokinomes”) provides novel adipokines, highlighting the critical roles of adipokines in relation to adipose tissue. In addition to secretome, the surface proteome (also termed surfaceome) of primary adipocytes derived from different mouse models for metabolic disorders was analyzed [66]. Because surface proteome also represents a cellular signaling gateway against the microenvironment, a comparative surface proteome analysis could reveal the functional contribution of adipocytes in metabolic disorders. Through extensive analysis, researchers suggested that an improvement of hypoadiponectinemia and β-adrenergic lipolysis are potential novel targets, which can be used to antagonize adipocyte malfunctions in obesity and consequently block metabolic disorders.

The WAT of obese subjects encountered with hypoxia as a result of poor vascularization. Under a hypoxic condition, cells undergo a change from oxidative phosphorylation to anaerobic energy metabolism, which influences lipid accumulation and adipocyte differentiation. Additionally, hypoxia leads to inflammation and cellular dysfunction and consequently induces the altered secretion of adipokines and insulin resistance [67,68]. Thus, the effect of hypoxia on the (pre)adipocyte secretome is one of the main interests in the field of adipocyte biology. Rosenow *et al.* analyzed the secretome of human preadipocytes and adipocytes after a treatment with the hypoxia-mimetic factor, CoCl_2_ [67]. The secretome changes were predominantly related to protein down-regulation and extracellular matrix protein dysregulation. Frazier *et al.* conducted a secretome analysis using adipose-derived stem cells (ASCs) from five female donors under low O_2_ culture conditions [68]. They indicated that the hypoxia condition in ASCs resulted in reduced extracellular matrix (ECM) protein and type II cytokine secretions that are significant with regard to inflammation.

### 3.3. Proteomic Means of Post-Translational Modifications in White Adipocytes

Protein post-translational modifications (PTMs) during adipogenesis have also been investigated. However, intensive studies using proteomic tools have not been done. There were only a few reports with regard to the importance of protein PTMs during adipogenesis via proteomic approaches. Our group has been interested in the involvement of protein tyrosine phosphatases (PTPs) during adipogenic differentiation. Therefore, we conducted PTP profiling studies of the white adipogenesis of 3T3-L1 cells and human mesenchymal stem cells [69,70,71,72]. The molecular mechanisms of several PTPs displaying differential expression patterns during adipogenesis were determined. Specifically, PTP-RQ, LAR and RPTPμ were suggested as negative or positive regulators of adipogenesis. Our group also reported the importance of protein acetylation during adipogenesis via proteomic approaches [73,74]. Based on an analysis of acetylome during the adipogenesis of 3T3-L1 cells, various proteins showing significant quantitative changes were identified. Of them, the acetylation of malate dehydrogenase 1 (MDH1) was found to induce the enzymatic activity of MDH1 significantly and subsequently to increase the intracellular NADPH levels such that adipogenesis is required as a reducing force [73]. Additionally, malate dehydrogenase 2 (MDH2) acetylation was shown to be affected by the cellular energy state and subsequently to regulate adipogenic differentiation [74]. Recently, Xu *et al.* reported acetylome data similar to that reported by our group [75]. They also indicated a wide-ranging temporal protein acetylation change during 3T3-L1 adipocyte differentiation. Recently, a new type of PTM was reported by Mannuel and Frizzell and Merkley *et al.* [76,77]. They detected protein succination during the adipogenesis of 3T3-L1 cells. However, more work is required to acquire the detailed mechanism and biological significance pertaining to this finding. Collectively, although various PTMs are closely related to adipogenesis, the necessary investigations have not been performed. Thus, more extensive proteomic approaches are needed to elucidate the detailed roles of PTMs during adipogenesis.

### 3.4. Proteomic Analysis of Adipocytes in Response to Stimuli

The effects of various stimuli on adipocyte biology or differentiation were also assessed by proteomic approaches. Rahman *et al.* reported the effect of chitosan oligonucleotides on 3T3-L1 adipocyte differentiation using 2-DE combined with MS [78]. Pal *et al.* reported the effects of rosiglitazone and guggulsterone during the adipogenic differentiation of 3T3-L1 cells via 2-DE combined with MS [79]. Rosiglitazone, a thiazolidinedione drug, is a potent insulin-sensitizing agent that enhances lipogenesis. On the other hand, guggulsterone is a natural drug extracted from the gum resin of *Commiphora mukul*, which induces apoptosis in 3T3-L1 cells. These two molecules show anti-diabetic effects; therefore, the protein list in this proteome screen may provide molecular insight into the action mode. Rosenow *et al.* analyzed the effect of resveratrol on the human adipocyte secretion profile [80]. Resveratrol, a natural polyphenolic compound, is known to mimic the beneficial effects of calorie restriction. To examine the effect of resveratrol on the secretion profile of human SGBS adipocytes, a 2-DE analysis was performed. The data clearly showed that resveratrol induces the secretion of proteins related to stress responses and apoptosis. Furthermore, the up-regulation of adiponectin and ApoE accompanied by the repressed expression of PAI-1 and PEDF secretion were found. These processes help to improve anti-inflammatory responses and enhance insulin sensitivity. Additionally, Joo *et al.* reported the effect of capsaicin on the rat WAT proteome through a 2-DE analysis [81]. Capsaicin, a main ingredient in hot peppers, shows anti-obesity activity by decreasing energy intake, the adipose tissue amount, and serum triglyceride levels via the stimulation of lipid mobilization. Researchers detected several proteins that were differentially expressed upon a treatment with capsaicin, with most of them related to thermogenesis or lipid metabolism. Kim *et al.* reported proteome changes upon a treatment with KR-62776, a PPARγ partial agonist [82]. They suggested that KR-62776 inhibits adipocyte differentiation via the activation of ERK. These proteomic analyses may provide the clues with which to understand the molecular mechanisms of various substances during adipogenesis or in adipocyte biology.

## 4. Proteomic Analysis of BAT, or Brown Adipocytes

Unlike WAT, BAT plays a critical role in energy expenditure. It mainly consists of brown adipocytes, which have a number of mitochondria, multilocular lipid droplets, and which strongly express uncoupling protein-1 (UCP-1). UCP-1 proteins, which are located in the inner-membrane of the mitochondria, uncouple ATP synthesis from the proton gradient and instead generate heat. BAT has been recognized as a major site of both cold- and diet-induced thermogenesis, especially in small rodents [83]. Recently, BAT was identified in human adults via positron-emission tomography (PET). Furthermore, the amount of BAT is inversely correlated with the body mass index (BMI) in human. Therefore, BAT is considered to be a natural anti-obesity organ and many research groups have become interested in BAT and brown adipocytes. However, extensive proteomic studies of BAT or brown adipocytes have not been done compared to the proteomic studies of WAT or white adipocytes.

### 4.1. Proteomic Analyses of BAT or Brown Adipogenesis

Choi *et al.* undertook a differential proteome analysis of BAT from lean and obese rats of both genders fed a high-fat diet using 2-DE combined MS to determine the importance of gender in BAT biology upon exposure to a high-fat diet [84]. The proteome data demonstrated the greater expression of various proteins involved in thermogenesis and fat oxidation as well as the weaker expression of proteins related to fat synthesis in female rats as compared to male rats, indicating the greater susceptibility of males to obesity when exposed to a high-fat diet. Our group investigated proteome changes during the adipocyte differentiation of mouse primary preadipocytes [85]. Among several proteins showing differentially expressed patterns, we examined the molecular mechanism of cofilin-1 during brown adipocyte differentiation. These results could bring new insight into the detailed mechanisms of brown adipocyte differentiation and may identify potential therapeutic targets for anti-obesity. Thus, proteomics studies of BAT or brown adipocytes have mostly attempted to understand its thermogenic properties as well as the molecular mechanisms of brown adipogenesis.

### 4.2. Mitochondrial Proteomics of BAT

Navet *et al.* suggested the mitochondria proteome plasticity of brown adipocytes in the rat in response to cold acclimation [86]. They demonstrated that several proteins involved in the major catabolic pathways including ATP synthase and mitofilin were increased. Forner *et al.* analyzed the proteome differences between white and brown fat mitochondria using the SILAC method. They demonstrate a substantially difference between them. In addition, white fat mitochondria express proteins that not only support anabolic functions but also degrade xenobiotics, indicating protective functions of WAT [87].

### 4.3. Proteomic Approaches of Post-Translational Modifications of Brown Adipocytes

Studies that analyze the PTMs of brown adipocytes and during brown adipogenesis are still in their early stages. Several reports have investigated the roles of the PTMs of brown adipocytes during brown adipogenesis [88,89,90,91]. Kruger *et al.* defined tyrosine-phosphoproteome in the insulin-signaling pathway using mass spectrometry in conjunction with phosphotyrosine immunoprecipitation and SILAC in differentiated brown adipocytes. After complete adipogenic differentiation, they used an insulin treatment and identified the proteins showing differential expression patterns. They found a total of 40 proteins as insulin-induced effectors and suggested cross-talk between insulin signaling and calcium signaling [88]. Our group undertook protein tyrosine phosphatase profiling using the HIB-1B brown preadipocyte cell line and mouse primary brown preadipocytes during brown adipogenesis [89,90]. Among the protein tyrosine phosphatases showing differential expression patterns, DUSP12 and DUSP10 were further analyzed to determine their functional roles in brown adipogenesis [90,91].

### 4.4. Comparative Proteomics of BAT and WAT

Comparisons of BAT and WAT at the proteome level have also been of great interest. Joo *et al.* reported differentially expressed proteins in WAT and in BAT by a proteomic analysis of obesity-susceptible and obesity-resistant rats fed a high-fat diet. They found various differentially expressed proteins in WAT and BAT. Thus, their proteomic results provide information about different propensity levels to obesity [92]. Okita *et al.* determined the differential responses of WAT and BAT to calorie restriction in rats using a 2-DE analysis [93]. Calorie restriction activates mitochondrial energy metabolism and fatty acid biosynthesis in WAT, indicating that WAT functions as an energy transducer, which forms glucose into an energy-dense lipid. On the other hand, calorie restriction induced either no effect or reduced the mitochondrial electron transport chain while enhancing fatty acid biosynthesis, suggesting that the function of BAT may be changed from an energy-consuming function to an energy reservoir function. Schmid *et al.* also reported the differential protein expression of BAT and WAT by a proteomic analysis in diet-induced obese mice [94]. They found that ubiquinol-cytochrome c reductase 1 (UQCR1) expression is increased in BAT and decreased in WAT, suggesting that this opposite regulation of UQCR1 in two types of adipose tissue reflecte the distinctive functions of BAT and WAT in obesity.

## 5. Conclusions and Future Perspectives

Adipose tissue is a major endocrine organ that plays an essential role in energy homeostasis. The excessive accumulation of fat in adipose tissue causes obesity. Obesity and obesity-associated metabolic disorders are a worldwide problem in modern societies. Thus, a deeper understanding of adipose tissue and adipocyte biology is critical issue to overcome obesity and its related diseases. In this review, we describe the recent progress in adipose tissue research and adipocyte biological studies that rely on proteomic approaches in an attempt to suggest more effective ways to address this issue. Many researchers have utilized proteomic approaches to elucidate the functions and characteristics of WAT and white adipocytes. On the other hand, extensive proteomic analyses of BAT and brown adipocytes have not been done thus far due to the relatively recent findings in adult humans. With regard to beige/brite adipocytes, there are no reports of novel biomarkers or characteristics using proteomic analyses. This may be due to the difficulty related to the enrichment of beige/brite cells, the lack of immortalized cell lines, and the deficiency of established novel markers. However, it is widely accepted that WAT browning as well as BAT activation may be important strategies in the treatment of obesity. Therefore, more extensive studies of browning and BAT biology in the proteomics field are necessary. We believe and hope that such work may someday introduce ideas or methods in the proteomics field for effective treatment of obesity and its related metabolic diseases.
